# The Risk Factors Predicting Suicidal Ideation Among Perinatal Women in Japan

**DOI:** 10.3389/fpsyt.2020.00441

**Published:** 2020-05-15

**Authors:** Chika Kubota, Toshiya Inada, Tomoko Shiino, Masahiko Ando, Maya Sato, Yukako Nakamura, Aya Yamauchi, Mako Morikawa, Takashi Okada, Masako Ohara, Branko Aleksic, Satomi Murase, Setsuko Goto, Atsuko Kanai, Norio Ozaki

**Affiliations:** ^1^Department of Psychiatry, National Center of Neurology and Psychiatry, Kodaira, Japan; ^2^Department of Psychiatry, Nagoya University Graduate School of Medicine, Nagoya, Japan; ^3^Department of Psychiatry and Psychobiology, Nagoya University Graduate School of Medicine, Nagoya, Japan; ^4^Division of Developmental Emotional Intelligence Research Center for Child Mental Development, University of Fukui, Fukui, Japan; ^5^Center for Advanced Medicine and Clinical Research, Nagoya University Graduate School of Medicine, Nagoya, Japan; ^6^Department of Psychiatry, Liaison Medical Marunouchi, Nagoya, Japan; ^7^Department of Obstetrics and Gynecology, Goto Setsuko Ladies Clinic, Nagoya, Japan; ^8^Graduate School of Education and Human Development, Nagoya University, Nagoya, Japan

**Keywords:** major depressive disorder, harm avoidance, postpartum depression, suicidal ideation, self-harm

## Abstract

**Introduction:**

The aim of the present study was to elucidate the foreseeable risk factors for suicidal ideation among Japanese perinatal women.

**Methods:**

This cohort study was conducted in Nagoya, Japan, from July 2012 to March 2018. The Edinburgh Postnatal Depression Scale (EPDS) questionnaire was conducted at four time points: early pregnancy, late pregnancy, 5 days postpartum, and 1 month postpartum. A total of 430 women completed the questionnaires. A logistic regression analysis was performed using the presence of suicidal ideation on the EPDS as an objective variable. The explanatory variables were age, presence of physical or mental disease, smoking and drinking habits, education, hospital types, EPDS total score in early pregnancy, bonding, and quality and amount of social support, as well as the history of major depressive disorder (MDD).

**Results:**

The rate of participants who were suspected of having suicidal ideation at any of the four time points was 11.6% (n=52), with the highest (n=25, 5.8%) at late pregnancy. For suicidal ideation, education level (OR: 1.19; 95% CI: 1.00–1.41; p=0.047), EPDS total points in the pregnancy period (OR: 1.25; 95% CI: 1.16–1.34; p < 0.000), a history of MDD (OR: 2.16; 95% CI: 1.00–4.79; p=0.049), and presence of mental disease (OR: 2.39; 95% CI: 1.00–5.70; p=0.049) were found to be risk factors for suicidal ideation. Age [odds ratio (OR): 0.88; 95% confidence interval (CI): 0.80–0.95; p=.002] and quality of social support (OR: 0.77; 95% CI: 0.60–0.99; p=.041) were found to be protective factors.

**Conclusion:**

Based on these results, effective preventive interventions, such as increasing the quality of social support and confirming the history of depression, should be carried out in pregnant depressive women at the early stage of the perinatal period.

## Introduction

Suicide prevention for expecting mothers is one of the most important problems in the field of perinatal mental health. A previous prospective cohort study reported that suicide was the leading cause of maternal death (8.7 per 100,000 women) in 2004–2015 in Tokyo, Japan (1). Maternal death rates (per 100,000 women) reported in countries other than Japan are as follows: 2.0 in the United States (2), 1.3 in Italy (3), 3.7 in Sweden (4), 5.9 in Finland (5), 2.6 in Canada (6), and 2.5 in the United Kingdom (7). Although the present survey is limited to Tokyo (1), the estimated suicide rate of perinatal women in Japan is higher than that in other countries.

Various psychosocial factors may be involved in maternal suicide. The following risk factors for maternal suicide have been reported: younger age (8, 9), unmarried (9), a history of family suicide, poverty (10), domestic violence (11), a history of abuse (12), racial issues (9, 13), regional isolation (9), anxiety (12, 14), suicidal ideation (15), a history of suicidal attempts (15), unexpected pregnancy (15), fetal and infant death (16), and mental disorders (14, 17, 18) such as major depressive disorder (MDD) (7, 8, 15, 19), bipolar disorder (19), and substance-related disorders (17).

In Japan, although general surveys on suicide have been widely conducted, research focusing on suicide among perinatal women is lacking. According to a Suicide Prevention Survey conducted in Japan in 2016 (20), young women had a high rate of experiencing suicidal ideation and suicide attempts. In that survey, the rates of women who had thoughts of suicidal ideation in their lifetime were reported to be 37.9 and 36.3% among those in their 20s and 30s, respectively, which translates to approximately 400,000 women among the total of 1,300,000 in that age range. In addition, about 200,000 women in their 20s and 30s have been estimated to have attempted suicide within the last year. According to a survey by the Ministry of Health, Labour and Welfare, the total number of suicides in 2018 among women in their 20s and 30s was 1,464 (21). About 40% of people who successfully committed suicide had attempted suicide at some point during their lifetime. Therefore, strategies such as introducing consultants for suicide prevention at a stage within suicidal ideation and providing psychosocial education for suicide prevention are frequently implemented in Japan. The need for similar activities for perinatal women has also been pointed out.

A number of studies have reported risk factors for suicidal ideation among perinatal women in countries other than Japan. A systematic review of 57 articles carried out by Gelaye et al. identified intimate partner violence, < 12 years of education, and MDD as risk factors for antenatal suicidal ideation (22).

A systematic review of 15 published research studies carried out by O'Connor et al. reported that women with a lower socioeconomic background and those who experience intimate partner violence are at increased risk for suicidal ideation (23). Ishida et al. examined the association between mental health problems in pregnant women and those in the postpartum period among 6,538 women aged 15–49 years in Paraguay, and reported that the risk for antenatal suicidal ideation was significantly higher when the pregnancy was unintended (24). They also reported that unintentionally pregnant women who had neither been in a union nor had a child were at a significantly higher risk for suicidal ideation compared with non-pregnant and non-postpartum women (24). Bodnar-Deren et al., who examined postnatal suicidal ideation among 1,073 mothers, reported that race/ethnicity, nativity, insurance, and language were significantly correlated with suicidal ideation at 3 weeks, 3 months, and 6 months postpartum (25). Sit et al. examined the associations between suicidal ideation among 628 depressed postpartum women and the following possible risk factors: experience of abuse as a child or adult, sleep disturbance, and anxiety symptoms (12). Sit et al. indicated that suicidal ideation among mothers was related to childhood physical abuse. They also reported that suicidal ideation among mothers with no history of childhood physical abuse was related to sleep disturbance and anxiety symptoms (12). Gelaye et al. also pointed out the urgent need for innovative approaches to improve the screening and detection of antepartum suicidal ideation, given that a substantial proportion of women with suicidal ideation do not meet the clinical thresholds for depression, and that the stress–diathesis model shows susceptibility to suicidal behavior independent of depressive disorders (22). Identifying the factors that cause suicidal ideation independent of depressive disorders is also desirable in Japan.

However, unfortunately, compared with death resulting from physical problems, suicide has not been emphasized as a reason for maternal death in Japan, and findings contributing to suicide prevention have been scarcely reported. Moreover, since suicide is affected by a country's economic status and cultural background, foreign survey results cannot be generalized to Japan.

We have been conducting a prospective cohort study on perinatal depression since 2004; we have found that approximately 32% of all participants show some depressive symptoms, as assessed by the Edinburgh Postnatal Depression Scale (EPDS) at any one of the following four time points: early pregnancy, late pregnancy, 5 days postpartum, and 1 month postpartum (26). Suicide has typically been strongly associated with a history of suicide attempts, and a strong association has been identified between suicide attempts and suicidal ideation (26).

The aim of the present study was to elucidate the foreseeable risk factors for suicidal ideation among perinatal women from the previous report to promote more effective suicide prevention measures.

## Method

### Design

The data in the present study were extracted from a prospective cohort study conducted in Nagoya, Japan from July 2012 to March 2018.

### Participants

Participants were recruited during early pregnancy in a maternity class for psychological education about pregnancy and birth at individual facilities. This maternity class was provided by medical staffs for the prevention and early detection of postpartum depression and other mental disorders. At the end of the program, the study protocol was introduced, and applicants were invited to participate voluntarily in the study.

The following four hospitals participated in this prospective cohort study: one general hospital (Nagoya Teishin Hospital), two obstetrics and gynecology hospitals (Kaseki Hospital and Royal Bell Clinic), and one university hospital (Nagoya University Hospital). In Japan, obstetrics and gynecology hospitals mainly treat uncomplicated pregnant women. Pregnant women with complications are usually introduced into general or university hospitals. Perinatal women with severe complications are followed up at university hospitals, and scheduled hospitalization for birth is often performed. By contrast, general hospitals with a neonatal intensive care unit deal with emergency births.

The eligibility criteria were as follows: 1) pregnant female aged ≥20 years, 2) ability to read and write Japanese, 3) attended a gynecological checkup at one of the four hospitals.

### Ethical Considerations

This study was approved by the ethics committees of Nagoya University Hospital. All study procedures were conducted in accordance with the Declaration of Helsinki and other relevant ethical guidelines. Written informed consent for participation was obtained from all participants.

### Measurements

**Figure 1:**
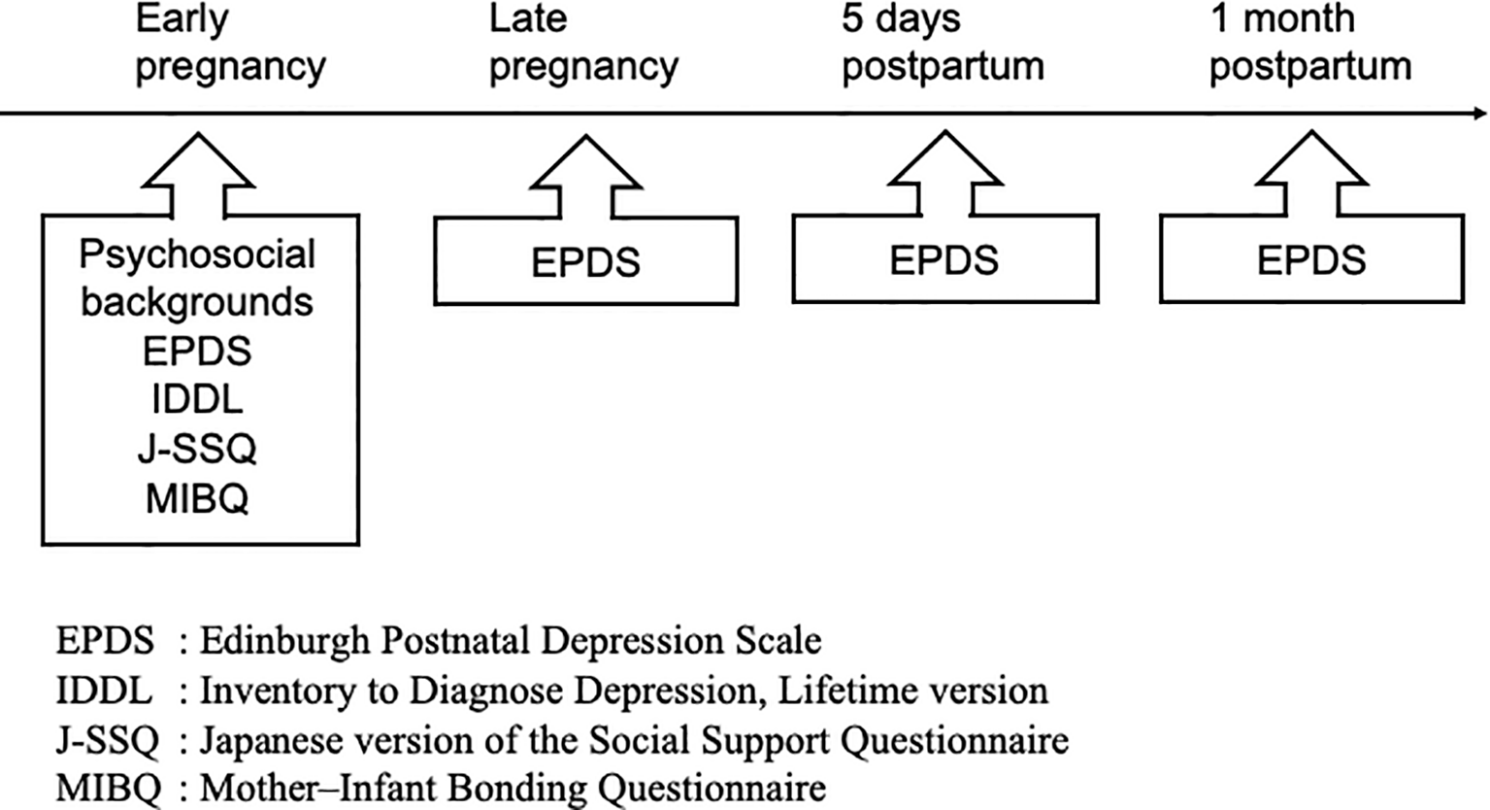
Flowchart of the study.

As shown in **Figure 1**, the participants' psychosocial backgrounds were evaluated at early pregnancy using a self-administered questionnaire with the following items: age, presence of physical and/or mental disease, smoking and drinking habits, years of schooling, number of childbirths, hospital types, bonding as assessed by the Mother–Infant Bonding Questionnaire (MIBQ), quality and amount of social support as assessed by the Japanese version of the Social Support Questionnaire (J-SSQ), and the presence of past depression from the Inventory to Diagnose Depression, Lifetime version (IDDL). In addition, the participants were asked to complete the EPDS at the following four time points: 1) early pregnancy, 2) late pregnancy, 3) 5 days postpartum, and 4) 1 month postpartum.

The presence or absence of physical and/or mental disease was obtained from the participants using the questionnaire. When the participants answered “present,” they were asked to provide further details, i.e., what kind of diseases they were suffering from. As for smoking and drinking habits, the participants were asked whether these habits were present or absent. When the participants answered “present,” they were also asked to provide further details, i.e., changes in frequency and volume before and after pregnancy. The hospital types were determined whether the hospital has a Neonatal Intensive Care Unit (NICU).

#### Edinburgh Postnatal Depression Scale

The EPDS is a self-administered questionnaire designed by Cox et al. in 1987 (27) to screen for postpartum depression. It is composed of 10 items scored on a four-point Likert scale. In our cohort study, we clarified that the Japanese version of the EPDS, which was established by Okano et al. in 1996 (28), had a three-factor structure of depression, anxiety, and anhedonia (29, 30). We adopted the evaluation of suicidal ideation based on previous reports (8, 12, 31). Participants who had a score of ≥2 on the 10th item of the EPDS during at least one of the four periods were classified into the suicidal ideation group; the remaining participants were classified into the healthy group. Howard et al. (8) examined the validity of evaluating suicidal ideation using the 10th item of the EPDS in a study involving 4,150 women at 6 weeks postpartum. In their study, the definition of suicidal ideation for the 10th item of the EPDS (a score of ≥2) was compared with that used in the Clinical Interview Schedule-Revised (CIS-R) (≥2 of five items on the CIS-R suicidal ideation measurement) (32). As a result, 79% of the participants who were classified as having suicidal ideation using the CIS-R were found to have a score of ≥2 on the 10th item of the EPDS, and a moderate kappa statistic value of 0.42 was observed between these two variables. It should be noted that the CIS-R has been validated across cultures and is widely used for the assessment of common mental disorders (33, 34).

#### Inventory to Diagnose Depression, Lifetime Version

The IDDL is a self-administered questionnaire that assesses the history of MDD in accordance with the Diagnostic and Statistical Manual of Mental Disorders (DSM)-III (35). The IDDL is composed of 22 items scored on a five-point Likert scale. A score of ≥3 indicates having a specific symptom for items 5 and 6, while a score of ≥2 indicates having a specific symptom for the remaining 20 items. All 22 items on the IDDL are classified into two major and seven other symptoms according to the DSM-III criteria for MDD. If one of the items indicating each symptom is over the cutoff score, the symptom is judged to be present. The criteria for having a history of MDD were as follows: 1) having the cutoff score or higher for five or more of the 22 items, and 2) these items contain one or more items of the two major symptoms. The sensitivity and specificity of the IDDL are 74 and 93%, respectively (35). The sensitivity and specificity of the Japanese version of the IDDL used in the present study, which was validated by Uehara et al., are 83 and 97%, respectively (36).

#### Japanese Version of the Social Support Questionnaire

The 12-item Social Support Questionnaire (SSQ-12) was developed to assess social support using a total of 12 questions for number and satisfaction subscales (37). The SSQ-12 is composed of six items for satisfaction level scored on a six-point Likert scale, and six items for the amount of social support. The SSQ-12 is a revised and shortened version of the original SSQ (38), which was composed of 27 items. The Japanese version of the SSQ-12 used in the present study was validated by Furukawa et al. (39).

#### Mother–Infant Bonding Questionnaire

Maternal positive feeling to their infant was called bonding. The MIBQ was developed to assess maternal bonding. It is composed of nine items rated on a four-point Likert scale (40). Higher scores mean a stronger negative feeling toward a child. We validated the Japanese version of the MIBQ in a previous cohort study (41). Prepartum negative feeling to the fetus as called bonding failure have been reported to predict postpartum negative feeling to the infant (42). Further, mothers with high suicidality have been reported to be less sensitive and responsive to their infants' cues (43). Therefore, bonding during pregnancy was evaluated.

### Statistical Analyses

With the presence or absence of suicidal ideation as an objective variable, this study was carried out using a logistic regression model with a forward selection procedure. The explanatory variables were: age, presence or absence of physical and/or mental disease, smoking and drinking habits, family income, years of schooling, EPDS total score in early pregnancy, bonding as assessed by the MIBQ, quality and amount of social support as assessed by the J-SSQ, and the presence of past depression as assessed by the IDDL. Listwise deletion methods were performed for missing values. The variance inflation factor (VIF) was calculated to evaluate multicollinearity. These explanatory variables were compared between groups based on the presence or absence of suicidal ideation. All statistical analyses were performed using IBM SPSS version 26.0 (IBM Japan, Tokyo, Japan.).

## Results

Of 457 participants enrolled at early pregnancy [mean (M): 5.3 months, SD: 1.6 months] in the present study, 430 perinatal women were finally included in the analysis. The mean age of the participants was 33.0 years [standard deviation (SD): 4.7 years]. The mean years of schooling was 15.0 (SD: 2.0). The rates of nulliparous, primiparous, and those who had given birth two or three times were 81.8, 15.8, 2.0, and 0.5%, respectively. The current drinking and smoking rates were 9.8 and 2.8%, respectively. The prevalences of mental and physical disorders were 16.2 and 38.9%, respectively. The rate of participants who were suspected of having suicidal ideation at any of four time points was 13.0% (5.5, 5.8, 2.3, and 4.4% at early pregnancy, late pregnancy, 5 days postpartum, and 1 month postpartum, respectively). The mean EPDS total scores at early pregnancy, late pregnancy, 5 days postpartum, and 1 month postpartum were 5.3 (SD: 5.0; range: 0–29), 4.9 (SD: 4.8; range: 0–30), 5.5 (SD: 4.9; range: 0–26), and 5.8 (SD: 5.2; range: 0–25), respectively. The mean IDDL total score was 30.4 (SD: 16.8; range: 0–81). The ratio of participants who were suspected of having a history of MDD based on the IDDL was 29.5% (n=127). The mean MIBQ total score was 3.5 (SD: 3.5; range: 0–17). The mean amount and quality of social support according to the J-SSQ were 3.8 (SD: 2.1; range: 0–16) and 4.8 (SD: 1.4; range: 0–6), respectively. A comparison between the two groups divided by the presence or absence of suicidal ideation is shown in **Table 1**.

**Table 1:** Comparison between groups (n=430) by presence or absence of suicidal ideation.

	Group 1 (n=379)	Group 2 (n=51)	p value
Age (y)	33.3 ± 4.6	31.6 ± 4.7	.013
Years of schooling	15.0 ± 1.9	15.3 ± 2.10	.250
Nulliparous Primiparous Given birth twice Given birth three times	82.3% 15.3% 1.8% 0.6%	78.4% 17.6% 4.0% 0%	.705
Drinking rate	9.0%	17.6%	.053
Smoking rate	1.3%	11.8%	<0.000
Prevalence of mental disorders	13.1%	44.9%	<0.000
Prevalence of physical disorders	39.0%	45.0%	.486
EPDS at early pregnancy	4.39 ± 3.95	11.55 ± 6.66	<0.000
EPDS at late pregnancy	4.11 ± 4.03	10.27 ± 6.64	<0.000
EPDS at 5 days postpartum	4.84 ± 4.32	9.73 ± 6.30	<0.000
EPDS at 1 month postpartum	5.15 ± 4.52	10.53 ± 7.13	<0.000
History of MDD	24.2%	54.9%	<0.000
IDDL	28.39 ± 15.65	45.20 ± 17.35	<0.000
MIBQ	3.19 ± 2.96	4.90 ± 5.37	.084
J-SSQ (support number)	3.95 ± 2.16	3.20 ± 1.73	.016
J-SSQ (satisfaction level)	4.90 ± 1.36	4.22 ± 1.35	.001

EPDS, Edinburgh Postnatal Depression Scale; MDD, major depressive disorder; IDDL, Inventory to Diagnose Depression, Lifetime version; MIBQ, Mother–Infant Bonding Questionnaire; J-SSQ, Japanese version of the Social Support Questionnaire

Data are presented as the mean ± standard deviation (SD).

A t-test was used to compare age, years of schooling, and J-SSQ scores.

The chi-squared test was used to compare the number of children, drinking, and smoking rates, prevalence of mental and physical disorders, and rate of having a history of MDD.

The Mann–Whitney U test was used to compare EPDS, IDDL, and MIBQ scores.

No explanatory variable was excluded because the VIF was <5. As shown in **Table 2**, education level (OR: 1.19; 95% CI: 1.00–1.41; p=0.047), EPDS total points in the pregnancy period (OR: 1.25; 95% CI: 1.16–1.34; p < 0.000), a history of MDD (OR: 2.16; 95% CI: 1.00–4.79; p=0.049), and presence of mental disease (OR: 2.39; 95% CI: 1.00–5.70; p=0.049) were found to be risk factors for suicidal ideation. Age [odds ratio (OR): 0.88; 95% confidence interval (CI): 0.80–0.95; p=.002] and quality of social support (OR: 0.77; 95% CI: 0.60–0.99; p=.041) were found to be protective factors.

**Table 2:** Forward stepwise logistic regression models.

Covariates	Model 1: full model			Model 2			Model 3			Model 4			Model 5			Model 6			Model 7			Model 8: final model		
	OR	95% CI	p Value	OR	95% CI	p Value	OR	95% CI	p Value	OR	95% CI	p Value	OR	95% CI	p Value	OR	95% CI	p Value	OR	95% CI	p Value	OR	95% CI	p Value
Age	0.89	0.81–0.97	0.008	0.89	0.81–0.97	0.008	0.89	0.81–0.97	0.008	0.89	0.81–0.97	0.008	0.89	0.81–0.97	0.006	0.89	0.81–0.97	0.006	0.88	0.81–0.96	0.005	0.88	0.80–0.95	0.002
Years of schooling	1.20	1.02–1.43	0.033	1.21	1.02–1.43	0.032	1.20	1.02–1.43	0.032	1.20	1.02–1.43	0.032	1.19	1.01–1.41	0.038	1.20	1.01–1.41	0.036	1.19	1.01–1.40	0.041	1.19	1.00–1.41	0.047
The presence or absence of mental disease	2.19	0.90–5.30	0.083	2.19	0.90–5.30	0.083	2.18	0.90–5.28	0.083	2.19	0.91–5.29	0.082	2.27	0.95–5.43	0.065	2.36	0.99–5.61	0.053	2.44	1.03–5.79	0.043	2.39	1.00–5.70	0.049
A history of MDD	2.26	1.01–5.08	0.048	2.25	1.01–5.04	0.048	2.26	1.01–5.05	0.046	2.26	1.01–5.05	< 0.001	2.24	1.01–4.98	0.048	2.22	1.00–4.93	0.049	2.22	1.00–4.92	0.049	2.16	1.00–4.79	0.049
Quality of social support	0.75	0.58–0.99	0.041	0.75	0.58–0.99	0.040	0.75	0.58–0.98	0.037	0.75	0.57–0.97	0.028	0.75	0.57–0.97	0.028	0.75	0.58–0.97	0.028	0.74	0.57–0.96	0.025	0.77	0.60–0.99	0.041
EPDS total point in the pregnancy period	1.25	1.15–1.35	< 0.00	1.25	1.15–1.35	< 0.000	1.25	1.15–1.35	< 0.000	1.25	1.16–1.35	< 0.000	1.26	1.17–1.36	< 0.000	1.26	1.17–1.36	< 0.000	1.26	1.17–1.36	< 0.000	1.25	1.16–1.34	< 0.000
Number of childbirths	0.57	0.23–1.39	0.216	0.58	0.24–1.38	0.216	0.58	0.24–1.39	0.219	0.58	0.24–1.39	0.221	0.55	0.23–1.30	0.172	0.55	0.23–1.30	0.173	0.55	0.23–1.31	0.178			
Drinking habits	1.65	0.49–5.58	0.420	1.63	0.49–5.38	0.425	1.63	0.49–5.40	0.421	1.66	0.51–5.47	0.403	1.90	0.63–5.78	0.257	1.93	0.63–5.87	0.247						
Hospital types	1.19	0.74–1.93	0.479	1.19	0.74–1.91	0.485	1.19	0.74–1.91	0.471	1.20	0.74–1.91	0.462	1.20	0.75–1.93	0.445									
Smoking habits	1.77	0.37–8.55	0.475	1.80	0.38–8.51	0.458	1.78	0.38–8.34	0.464	1.74	0.37–8.19	0.481												
Amount of social support	0.97	0.79–1.20	0.781	0.97	0.79–1.20	0.768	0.97	0.79–1.20	0.767															
MIBQ total points in the pregnancy period	1.01	0.92–1.11	0.863	1.01	0.91–1.11	0.866																		
The presence or absence of physical disease	0.95	0.44–2.01	0.904																					

EPDS, Edinburgh Postnatal Depression Scale; MDD, major depressive disorder.

## Discussion

The present study investigated the risk factors for suicidal ideation among perinatal women in Japan. The results indicated that higher education, more severe depressive symptoms in early pregnancy, a history of MDD, and presence of mental disease were significant risk factors, and that age and social support were significant protective factors.

Since the rate of suicidal ideation in early and late pregnancy was high, early intervention from the prenatal period is important. In the present study, the most important risk factors for suicidal ideation in the perinatal period in Japan were current and previous depressive symptoms.

In previous studies, age (8, 44) and social support (45) were reported to be protective factors that reduce suicidal ideation in pregnant women. However, since other confounding factors, such as economic status and unwanted pregnancies, may exist in the background, future research is needed to identify the exact role of these factors.

Meanwhile, in contrast to the present results, an association has been reported between fewer years of education and a higher rate of maternal suicide (46). In Japan, highly educated women are often forced to leave their jobs after childbirth because they cannot receive sufficient support to balance work and childbearing. The present results may reflect this situation.

This study had several limitations. First, it could have involved a selection bias because individuals who did not have any interest in mental health or a history of depressive illness may not have responded to the questionnaire, and therefore, would have been excluded from the analysis. In fact, the rate of participants who had a history of MDD as measured by the IDDL was quite high compared with previous reports (35, 36). Second, information regarding the presence or absence of mental illness was obtained from the questionnaire responses. Third, the definition of suicidal ideation in this study was based on that used in previous reports, as opposed to clinical diagnostic interviews by psychiatrists; such interviews should be conducted in future research.

In the present study, the rate of perinatal suicidal ideation was 13.0%. To our knowledge, this is the first study to investigate the rate of perinatal suicidal ideation in Japan. In previous reports from overseas, the rate of suicidal ideation during pregnancy has ranged from 2.6 to 22.8% (47–49), and that during the postpartum period from 6.16 to 14% (19, 49). The present result regarding the rate of perinatal suicidal ideation of 11.6% is within the range of these previous reports. This rate can vary greatly depending on the country, cultural differences, and evaluation methods. Since pregnancy and childbirth are not protective factors for suicidal ideation, further research is needed to help prevent suicide in perinatal women.

Based on the results of this study, effective preventive interventions, such as increasing the quality of social support and confirming the history of depression, should be carried out in pregnant depressive women at the early stage of the perinatal period.

## Data Availability Statement

The datasets generated for this study are available on request to the corresponding author.

## Ethics Statement

The studies involving human participants were reviewed and approved by the ethics committees of Nagoya University Hospital. The patients/participants provided their written informed consent to participate in this study.

## Author Contributions

SM, SG, AK, and NO conceived and designed the experiments. CK, YN, AY, TS, MM, MO, MS, and TO performed the experiments. CK, MA, and NO conducted the statistical analysis. CK, TI, BA, and NO wrote the paper. All authors contributed to and have approved the final manuscript.

## Funding

Funding for this study was provided by research grants from the Ministry of Education, Culture, Sports, Science and Technology of Japan; the Ministry of Health, Labour and Welfare of Japan; The Academic Frontier Project for Private Universities, Comparative Cognitive Science Institutes, Meijo University; the Core Research for Evolutional Science and Technology; Intramural Research Grant (21B‐2) for Neurological and Psychiatric Disorders from the National Center of Neurology and Psychiatry; the Specific Research Fund 2012 for East Japan Great Earthquake Revival by The New Technology Development Foundation, and the Japan Agency for Medical Research and Development; the Research and Development Grants for Comprehensive Research for Persons with Disabilities from Japan Agency for Medical Research and development, AMED (No. JP18dk0307077).

## Conflict of Interest

The authors declare that the research was conducted in the absence of any commercial or financial relationships that could be construed as a potential conflict of interest.
